# Identification of *Trichoderma* spp., Their Biomanagement Against *Fusarium proliferatum*, and Growth Promotion of *Zea mays*

**DOI:** 10.3390/jof11090683

**Published:** 2025-09-19

**Authors:** Eman G. A. M. El-Dawy, Youssuf A. Gherbawy, Pet Ioan, Mohamed A. Hussein

**Affiliations:** 1 Botany and Microbiology Department, Faculty of Science, South Valley University, Qena 83523, Egypt; youssuf.gherbawy@svu.edu.eg (Y.A.G.); m.hussein@sci.svu.edu.eg (M.A.H.); 2 Applied and Environmental Microbiology Center, South Valley University, Qena 83523, Egypt; 3 Biotechnology Department, Faculty of Bioengineering, University of Life Sciences “King Mihai I”, 300645 Timisoara, Romania

**Keywords:** *Trichoderma* spp., phylogenetic characterization, biocontrol agents, maize growth promotion, fungal pathogens

## Abstract

Species of *Trichoderma* are currently in high demand as eco-friendly and commercial biocontrol agents due to the proliferation of organic farming methods. This study focused on the potential biocontrol agents of *Trichoderma* against plant-pathogenic fungi. *Trichoderma* strains were isolated from different sources (soil, grapevine tissues, lemon fruit, and maize seeds), and were characterized morphologically on two culture media, i.e., Potato Dextrose Agar and Malt Extract Agar, and molecularly using two gene regions: translation elongation factor 1 (TEF) and nuclear ribosomal internal transcribed spacer (ITS). Phylogenetic trees were constructed. As a result, two *Trichoderma* species were identified, i.e., *T. afroharzianum* and *T. longibrachiatum.* The biocontrol effects of all isolated strains of *Trichoderma* on *Fusarium* plant damping-off and the promotion of plant growth were evaluated. Additionally, the antagonistic efficiency of *Trichoderma* spp. against *F*. *proliferatum* using the dual-culture method was evaluated. Under greenhouse conditions, *T*. *afroharzianum* strains AEMCTa3 and AEMCTa6 were used to treat maize plants infected with *Fusarium*. The application of *Trichoderma* significantly reduced the disease index to 15.6% and 0%, respectively. Additionally, maize seedlings showed significant improvements in shoot and root lengths and fresh and dry weights and increased photosynthetic pigment contents compared to *Fusarium*-infected plants and the untreated control. The gas chromatography–mass spectrometry (GC-MS) analysis of *T. afroharzianum* extracts identified a variety of bioactive compounds. These compounds included antifungal substances like N-ethyl-1,3-dithioisoindoline, as well as plant growth-promoting hormones like 6-pentyl-α-pyrone and gibberellic acid. Interestingly, the analysis also revealed new phenylacetic acid derivatives that may play important roles in both plant health and disease resistance. From a practical perspective, developing diverse application methods for *Trichoderma* is essential to optimize its role as a biocontrol agent and a plant growth promoter, thereby supporting sustainable agriculture through improved adaptability and effectiveness across different farming systems.

## 1. Introduction

*Fusarium proliferatum* is a globally distributed phytopathogen that poses a major threat to maize production, causing ear and stalk rot and leading to substantial yield losses. Additionally, it produces mycotoxins called fumonisins, which are harmful to both human and animal health and are a major concern for food safety and commerce. The necessity of developing efficient biocontrol methods against this fungus is underscored by its high frequency in maize-growing areas and its dual impact on crop quality and yield [1]. *Trichoderma* has gained significant attention as a biological control agent, especially for its proven effectiveness and safety in combating diseases caused by *Fusarium* species [2].

The genus *Trichoderma* (Ascomycota, Sordariomycetes, Hypocreales, Hypocreaceae) was established by Persoon in 1794 with four initially described species [3]. Taxonomic revisions based on morphological and molecular data have confirmed that only *T. viride* remains in this genus. To date, approximately 500 species have been identified and documented [4]. Among these, *T. harzianum* has been recognized as a polyphyletic group, leading to its classification as a species complex. Using translation elongation factor 1-alpha (EF1α) sequences, this complex was divided into 14 distinct species [5,6]. Additionally, *T. longibrachiatum* group was revised by Samuels et al. [7], resulting in the recognition of 21 species. *Trichoderma* fungi are remarkably versatile, surviving in a wide range of ecological environments. Their diverse metabolism enables them to break down numerous substrates and produce numerous secondary metabolites, around 370 distinct compounds, many of which have antagonistic properties that play a vital role in protecting plant health [8].

Biological control agents (BCAs), including bacteria, viruses, fungi, insects, mites, nematodes, yeasts, and protozoa, fight plant pathogens through various biological mechanisms [9]. *Trichoderma* spp. produce hydrolytic enzymes such as β-1,3-glucanase and chitinase, which play a key role in degrading the cell walls of phytopathogenic fungi. These enzymatic activities are notably enhanced in the presence of pathogens, indicating an adaptive response that strengthens their biocontrol potential [10]. *Trichoderma* spp. not only suppress phytopathogens through direct antagonism but also enhance plant immunity. Their interaction with plant roots can trigger defense responses via pattern recognition receptors (PRRs), which detect conserved microbial signals known as pathogen- or microbe-associated molecular patterns (PAMPs/MAMPs). This recognition leads to the production of reactive oxygen species (ROS), activation of defense-related pathways, and transcription of pathogenesis-related (PR) genes, contributing to systemic acquired resistance (SAR) [11]. Upon encountering pathogens, BCAs activate genetic pathways to produce compounds that suppress disease and pathogen dominance [12]. Due to their diverse action methods, various species of the genus *Trichoderma* have been extensively studied and used as biological control agents in agriculture. Species of *Trichoderma* parasitize phytopathogenic fungi and plant-parasitic nematodes, producing potent biocidal components with antifungal, antibacterial, oomyceticidal, insecticidal, and nematicidal properties [13]. They effectively compete for space and nutrients in the rhizosphere, obstructing access to soil pathogens [14]. They also induce a local or systemic defensive response in host plants against potential future pathogen or pest attacks [15]. Moreover, various *Trichoderma* species function as plant growth-promoting fungi or biofertilizers by synthesizing and releasing plant hormones (cytokinins and auxins), solubilizing soil nutrients (such as potassium and phosphorus), or producing siderophores for metal chelation (including magnesium, iron, zinc, and copper) [15]. Ultimately, many *Trichoderma* species may enhance resistance to abiotic stressors, including drought, salt, and severe temperatures, via their interaction with the plant [16]. These action methods have resulted in commercial *Trichoderma* formulations constituting 50–60% of the global biofungicide industry and acting through multiple mechanisms, like mycoparasitism, antibiosis, competition, and induction of plant defense [17].

This study aimed to isolate *Trichoderma* spp. from different substrates, to characterize these isolates using morphological and molecular tools, and to evaluate their biocontrol potential against the globally distributed toxigenic pathogen *Fusarium proliferatum*, which affects yield and produces harmful toxins, and assess their ability to promote maize growth.

## 2. Materials and Methods

### 2.1. Sample Collection

The samples of soil (4), grapevine tissues (3), lemon fruit (2), and maize seeds (12) were randomly collected from different locations in Upper Egypt (no. = 18) and Italy (no. = 3) to ensure a representative diversity of *Trichoderma* species. This random sampling step aimed to capture a broad ecological range of the genus across different substrates. They were placed in sterile polystyrene bags to maintain sample integrity and were immediately transferred to the mycology laboratory to be stored at 4 °C until used for further isolation and identification of *Trichoderma* species.

### 2.2. Isolation of Trichoderma and Fusarium proliferatum

*Trichoderma* spp. were isolated on Potato Dextrose Agar (PDA, SRL) medium using a simple dilution-plate technique [18]. A 0.5 g quantity of the sample was diluted in 100 mL of sterilized H_2_O with hand shaking for 2 min. One milliliter of the resulting suspension was spread onto PDA to isolate the fungi. All *Trichoderma* spp. growing on PDA were isolated and purified with monosporic cultivation into pure cultures. *Fusarium proliferatum* was selected as the target pathogen because it was isolated from rotting roots of *Zea mays* (maize) plants collected in Qena City, Egypt.

### 2.3. Identification of Trichoderma Isolates

The *Trichoderma* strains were grown on PDA and MEA (SRL) at 28 °C for one week. The colony morphology (color, texture, and structure) and microscopic characteristics (conidia size and shape; phialides shape; and chlamydospores formation) for each *Trichoderma* isolate were recorded. The key provided by Siddiquee [19] was used.

### 2.4. DNA Extraction, PCR Amplification, and Sequencing Analysis

A modified method employing cetyltrimethylammonium bromide (CTAB, Sigma Aldrich, St. Louis, MO, USA) was used to extract genomic DNA [20]. Specifically, the incubation period was increased to one hour, which increased the effectiveness of cell lysis. The fungal identification was performed based on the sequence analysis of ITS using the universal primers (ITS1/ITS4: 3′-5′: CTTGGTCATTTAGAGGAAGTAA and TCCTCCGCTTATTGATATGC) [21] and the translation elongation factor 1 gene (tef-1α) (Ef728M/TefR1: CATCGAGAAGTTCGAGAAGG and TACTTGAAGGAACCCTTACC) [22,23]. The conditions for PCR amplification were described by Tomczyk et al. [24]. PCR products were purified and sequenced at Macrogen (Seoul, Republic of Korea). The obtained sequences were deposited in GenBank, and accession numbers were obtained and inserted into the phylogenetic trees.

DNA sequences were initially aligned using Clustal X version 1.81 [25]. Phylogenetic analysis was performed by applying the UPGMA (Unweighted Pair Group Method with Arithmetic Mean) algorithm as described by Sneath and Sokal [26], with genetic distances calculated using the Jukes–Cantor substitution model [27]. Additionally, the data were subjected to computer-assisted analysis using TREECON for Windows (version 1.3b, 1998), which generated a phylogenetic tree based on Maximum Likelihood and the neighbor-joining method [28].

### 2.5. In Vitro Evaluation of Trichoderma Antagonists Against Fusarium proliferatum

The efficacy of each *Trichoderma* isolate in inhibiting *Fusarium proliferatum* was examined via the dual-culture method, with three replicates [29]. The inhibition percentage (I%) of mycelial growth in *F. proliferatum* was determined using the following formula: I% = (r1 − r2)/r1 × 100, where r1 represents the radial growth of *F. proliferatum* in the control, and r2 denotes the radial growth of *F. proliferatum* in the dual-culture plate.

### 2.6. Greenhouse Experiment of Trichoderma on Maize Plants

A pot culture experiment was conducted to evaluate the antagonistic potential of *T. afroharzianum* AEMCTa3 and AEMCTa6 against *F. proliferatum.*

#### 2.6.1. Preparation of Fungal Inoculum

Wheat seeds were used as a substrate to prepare fungal inoculum. First, the seeds were soaked in distilled water for three hours. Then, 100 g of the seeds were transferred to conical Erlenmeyer flasks and autoclaved. The sterilized flasks were inoculated with actively growing disks of *F. proliferatum*, *T*. *afroharzianum* AEMCTa3, and AEMCTa6 cultures and incubated at 28 °C for ten days until the complete growth of the fungus [30].

#### 2.6.2. Plant Treatments and Growth Conditions

Seeds of maize (*Zea mays*) were obtained from the Agronomy Department, Faculty of Agriculture, South Valley University. Pots (20 cm in diameter) were sterilized by immersing them in a 5% formalin solution for 10 min and then letting them dry. The dried pots were filled with sterilized sandy-clay (1:2) soil mixed with fungal inoculum (inoculated wheat seeds) according to the treatments (T1: soil without fungal inoculum; T2: soil with 0.2% *T. afroharzianum* AEMCTa3; T3: soil with 0.2% *T. afroharzianum* AEMCTa6; T4: soil with 1% *F. proliferatum*; T5: soil with 1% *F. proliferatum* + 0.2% *T. afroharzianum* AEMCTa3; and T6: soil with 1% *F. proliferatum* + 0.2% *T. afroharzianum* AEMCTa6). Six seeds were sown in each pot, with three replicates conducted for each treatment. The pots were watered to field capacity and kept in a greenhouse with natural sunlight (12 h light/12 h night) at 28–30 °C during the day and 15–20 °C at night, with a humidity of 60–70% RH.

Disease incidence was estimated as the percentage of damping-off after 40 days of seed sowing [31]. Damping-off% = (no. of nonemerged seed/no. of sown seeds) × 100. Growth parameters of maize seedlings were also determined, including shoot and root lengths (measured using a standard ruler from the base to the tip of the respective organs), fresh and dry weights (the fresh weight was recorded immediately after harvesting using a digital balance, and the dry weight was measured after drying the plant samples in an oven at 70 °C for 48 h until a constant weight was achieved), and photosynthetic pigments (chlorophylls a and b and carotenoids were extracted using 80% acetone and quantified spectrophotometrically at specific wavelengths, i.e., 645 nm, 663 nm, and 470 nm).

### 2.7. Gas Chromatography–Mass Spectrometry (GC-MS) Analysis of Secondary Metabolites Produced by T. afroharzianum AEMCTa3 and AEMCTa6

The GC-MS analysis was performed on filtrates of two *T. afroharzianum* isolates (AEMCTa3 and AEMCTa6) obtained after their incubation in the PDB medium for two weeks at 28 °C. For each isolate, three replicates were analyzed. All detected compounds and their derivatives were identified. The average of relative abundance of each compound was determined, and descriptive statistics were used to summarize the data, including retention times and percentage composition.

The gas chromatography–mass spectrometry (GC-MS) analysis was performed using a Thermo Scientific TRACE GC Ultra™ equipped with a split/splitless injector and coupled to a Polaris Q quadrupole ion trap mass spectrometer (Thermo Electron, Waltham, MA, USA). A VB5 fused silica capillary column (30 m × 0.25 mm i.d., 0.25 μm film thickness; 5% phenyl, 95% methylpolysiloxane; J&W Scientific, Fisons, Folsom, CA, USA) was used. The injector and interface temperatures were set at 250 °C and 300 °C, respectively. The oven temperature was programmed from 50 °C to 250 °C at 4 °C/min and held for 3 min. Helium served as the carrier gas at a flow rate of 1 mL/min. A 1 μL sample was injected in the split mode (1:20). Mass spectrometry conditions included electron ionization (EI) at 70 eV and a scan range of 10–350 amu. Fungal extract components were identified by comparing their retention times and mass spectra with those of authentic standards and database entries [32].

### 2.8. Root Preparation and Histological Observations

Tissue dehydration, embedding, sectioning, and staining were performed as described by Livingston et al. [33]. The prepared slides of corn root tissues were examined under a light microscope (Leica DM1000, Leica, Wetzlar, Germany) equipped with a digital camera for photomicrography at 10× magnification. The histological observations of root tissue samples were made from four distinct sections of each plant.

### 2.9. Statistical Analysis

For the in vitro antagonism test: Data normality was assessed using the Shapiro–Wilk test, and homogeneity of variance was evaluated using Levene’s test. SPSS version 20 was used to perform the statistics on the collected data (SPSS Inc., Chicago, IL, USA). Duncan’s test and one-way analysis of variance (ANOVA) (at *p* < 0.05) were performed. The isolates AEMCTa3 and AEMCTa6 showed 100% inhibition and completely overgrew the *Fusarium* colony, so they were selected for further testing.

For the greenhouse test: The statistics of the obtained data were carried out using SPSS version 23 (SPSS Inc., Chicago, IL, USA). One-way analysis of variance (ANOVA) and Duncan’s test were used, and *p* < 0.05 was significant for the differences in the tested growth parameters and the concentration of photosynthetic pigments among treatments. Data normality was assessed using the Shapiro–Wilk test, and homogeneity of variance was evaluated using Levene’s test.

For the GC-MS analysis: Data normality and homogeneity of variance were assessed using the Shapiro–Wilk test and Levene’s test. All data were analyzed at the Analytical Chemistry Unit of Assiut University, Faculty of Science.

## 3. Results

### 3.1. Morphological Identification of Trichoderma Isolates

**Figure 1:**
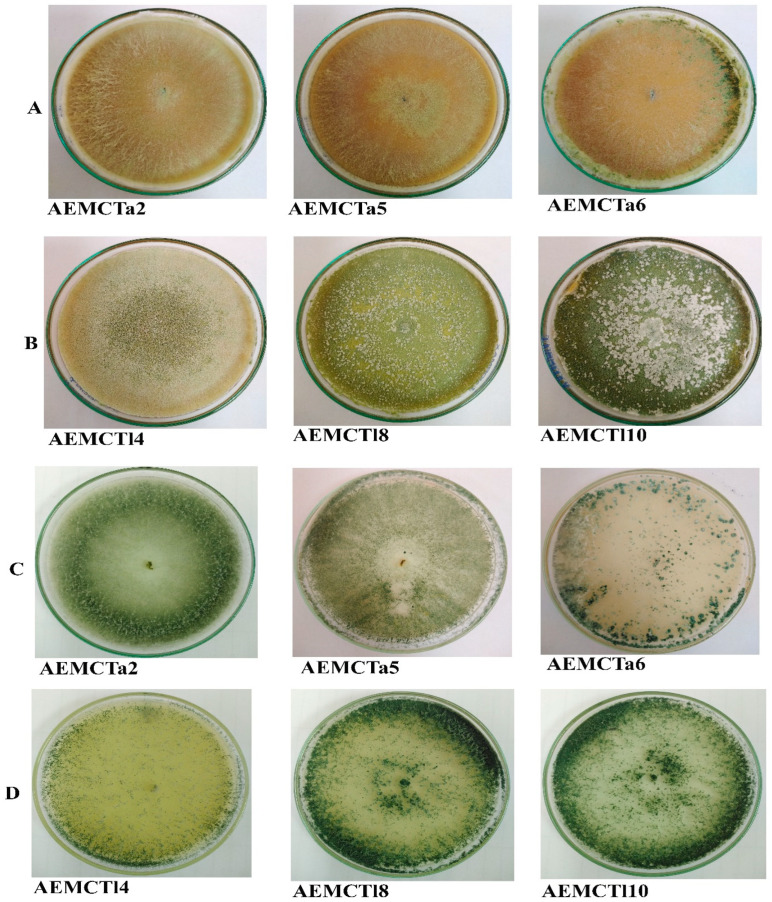
Colony features of *Trichoderma* spp. after incubation period of seven days at 28 °C: (**A**) *T. afroharzianum* on MEA medium; (**B**) *T. longibrachiatum* on MEA medium; (**C**) *T. afroharzianum* on PDA medium; and (**D**) *T. longibrachiatum* on PDA medium.

**Figure 2:**
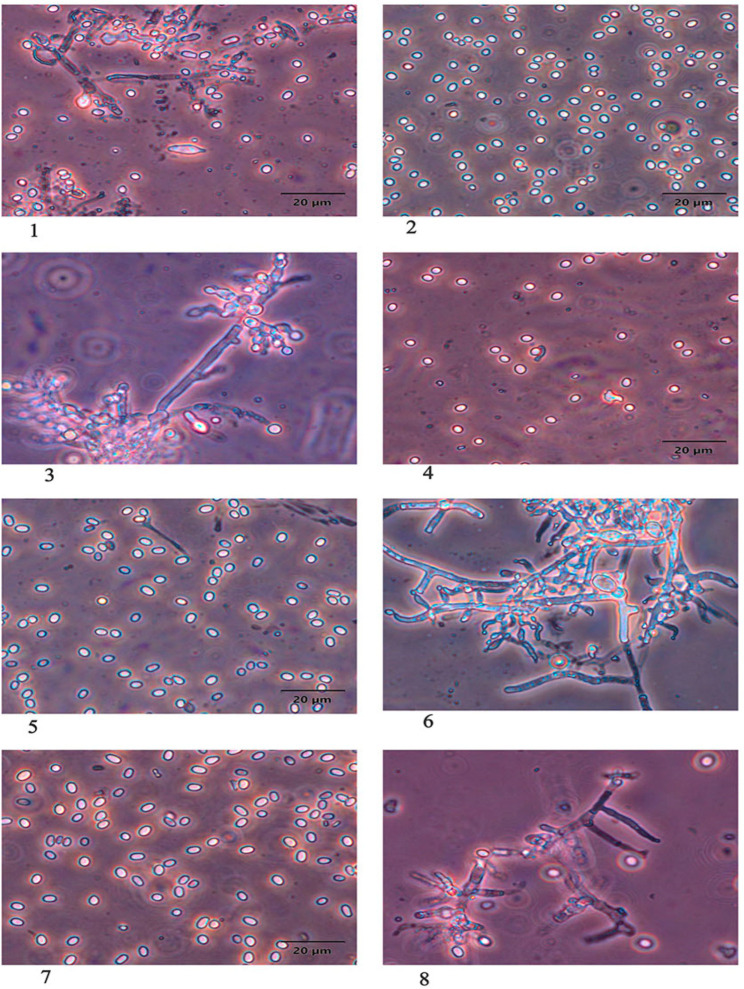
Microscopic characteristics (mycelium, phialides, and conidia) of *Trichoderma afroharzianum* and *T. longibrachiatum* cultured on PDA and MEA media after 7 days of incubation at 28 °C. Images of (**1**,**2**) *T. afroharzianum* on PDA; (**3**,**4**) *T. afroharzianum* on MEA; (**5**,**6**) *T. longibrachiatum* on PDA; and (**7**,**8**) *T. longibrachiatum* on MEA. All images captured at 40× magnification; scale bar = 20 µm.

The morphological identification of *T. afroharzianum* and *T. longibrachiatum* was supported by distinct characteristics observed on the PDA and MEA media. Both species exhibited characteristic colony pigmentation and conidial morphology, with few variations. *T. afroharzianum* was distinguished by its greenish conidial ring and dark brown reverse pigment, with sparse, floccose aerial mycelium. Microscopically, it showed highly branched conidiophores, ampuliform phialides, and predominantly ovate-to-globose conidia. Slight differences in conidial size and chlamydospore presence were noted between PDA and MEA. *T. longibrachiatum* displayed smooth, watery white colonies that produced dense greenish-yellow conidia and yellow-to-brown reverse pigmentation. It was characterized by long conidiophores, lageniform phialides, and ellipsoidal-to-ovate conidia. Chlamydospores were absent on both media. Notably, conidial size was slightly larger on MEA. These morphological features are summarized in Table 1 and illustrated in Figure 1 and Figure 2.

### 3.2. Molecular Identification of Trichoderma Isolates

**Figure 3:**
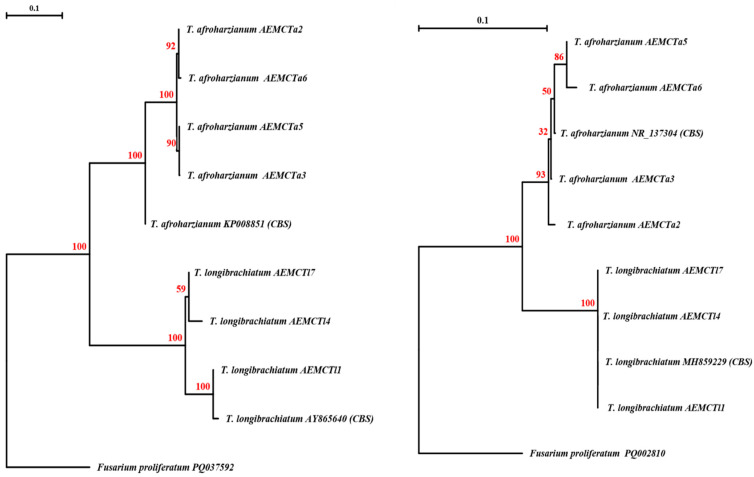
Phylogenetic trees constructed using the neighbor-joining method for *Trichoderma* spp. strains, based on the ITS gene sequence data (**left**) and tef gene sequence data (**right**). The numbers above the branches indicate bootstrap values, calculated after 1000 replications. Type strain sequences, with their GenBank accession numbers (CBS), are included in the phylogenetic trees. *Fusarium proliferatum*, isolated from maize root in this study, was used as the outgroup.

According to the ITS phylogenetic tree (Figure 3, left), the *T. afroharzianum* isolates AEMCTa2 (PQ002804), AEMCTa6 (PQ002808), AEMCTa5 (PQ002807), and AEMCTa3 (PQ013073) were grouped together in a clade with the reference strain *T. afroharzianum* KP008851 (CBS). The grouping was highly supported by bootstrap values of 92% (AEMCTa2–AEMCTa6), 100% (AEMCTa5–AEMCTa3), and 90–100% for major internal branches. For *T. longibrachiatum*, the isolates AEMCTl7 (PQ002809), AEMCTl4 (PQ002806), and AEMCTl1 (PQ002803) were grouped with the type strain AY865640 (CBS), with a bootstrap support of 100%. The outgroup *Fusarium proliferatum* PQ002810 was used, isolated from maize root in this study.

In the tef gene phylogenetic tree (Figure 3, right), the *T. afroharzianum* isolates AEMCTa5 (PQ037589), AEMCTa6 (PQ037590), AEMCTa3 (PX111767), and AEMCTa2 (PQ037586) were clustered with the reference strain NR_137304 (CBS), receiving varied bootstrap support (32–100%). For *T. longibrachiatum*, AEMCTl7 (PQ037591), AEMCTl4 (PQ037588), and AEMCTl1 (PQ037585) were grouped with the reference sequence MH859229 (CBS), supported by a bootstrap value of 100%. The outgroup in this analysis was *Fusarium proliferatum* PQ037592, which was also isolated from maize root.

### 3.3. Antagonism Efficiency of Trichoderma Species

**Figure 4:**
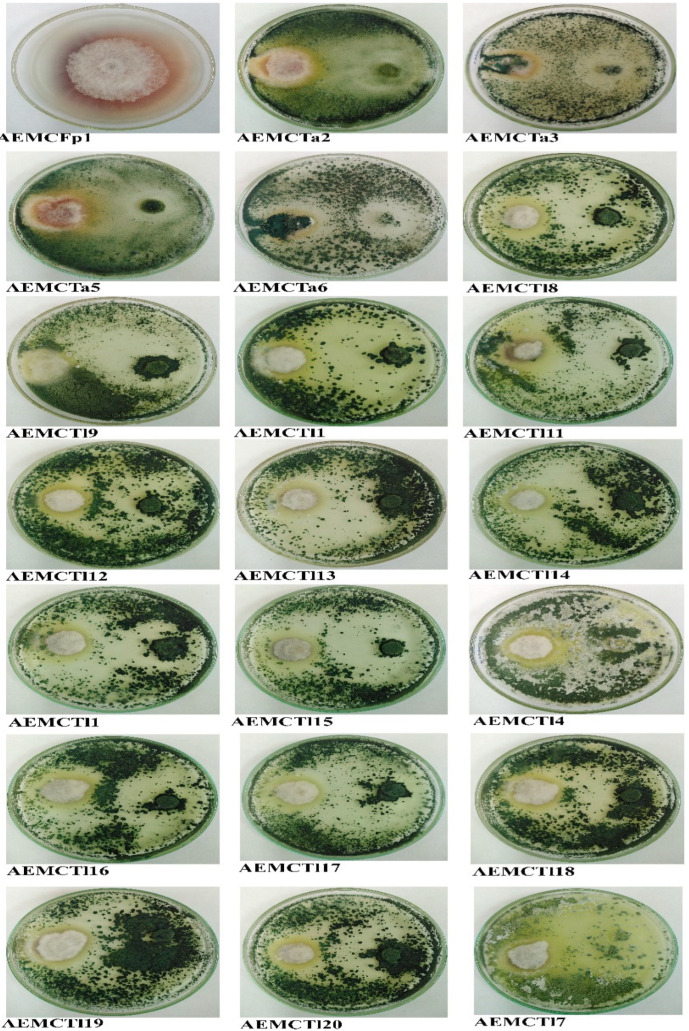
Antagonistic activity of *Trichoderma* strains against *Fusarium proliferatum* on PDA medium.

When *T. afroharzianum* (or *T. longibrachiatum*) and the pathogen *F. proliferatum* were cultivated in dual-culture plates, the growth of the pathogen was significantly inhibited. In the dual-culture plates, *F*. *proliferatum* grew much more slowly than in the control plates, which contained only a single culture incubated under the same conditions and for the same period. The contact zone looked curved in all the dual-culture plates, with the concavity toward *F. proliferatum*. The growth rate of each colony determined how the biocontrol agent colony and the pathogenic fungal colony developed radially on the same PDA plate. Every isolate of *Trichoderma* grew more quickly than *F. proliferatum*, a primary sign of a good biological agent. All twenty isolates of *Trichoderma* significantly inhibited the growth of *F. proliferatum* with ranges of 47.65 to 100%. It was noticed that the percentage of inhibition shown by isolates of AEMCTa3 and AEMCTa6 was 100%, and their hyphal mycelium had extended growth over the *Fusarium* colony. Thus, we have chosen the two isolates of AEMCTa3 and AEMCTa6 for further tests (Table 2, Figure 4).

### 3.4. Cultivation of Maize Plants Grown in Soil Inoculated with Fusarium proliferatum and Phytoremediation Using Trichoderma Strains

#### 3.4.1. Disease Suppression in Maize by *T. afroharzianum*

**Figure 5:**
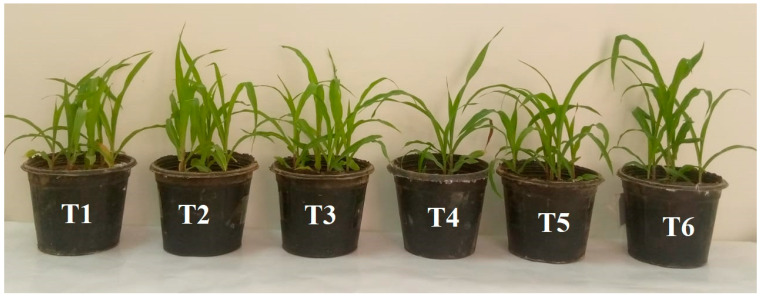
Maize plants grown on soil mixed with *F*. *proliferatum* and *T*. *afroharzianum*; T1: soil free from fungal inoculum; T2: soil inoculated with *T*. *afroharzianum* AEMCTa3; T3: soil inoculated with *T*. *afroharzianum* AEMCTa6; T4: soil inoculated with *F*. *proliferatum*; T5: soil inoculated with *F*. *proliferatum* + *T*. *afroharzianum* AEMCTa3; T6: soil inoculated with *F*. *proliferatum* + *T*. *afroharzianum* AEMCTa6.

*F. proliferatum* (T4) caused 50% damping-off, indicating its high aggressiveness and pathogenicity on maize seedlings. When co-inoculated with *T. afroharzianum* AEMCTa3 (T5), the disease incidence dropped to 16.66%, showing a strong biocontrol effect. *T. afroharzianum* AEMCTa6 (T6) completely suppressed the disease (0% damping-off), indicating superior antagonistic activity compared to AEMCTa3 (Table 3, Figure 5).

#### 3.4.2. Fresh and Dry Weights and Lengths of Maize Shoots and Roots

**Figure 6:**
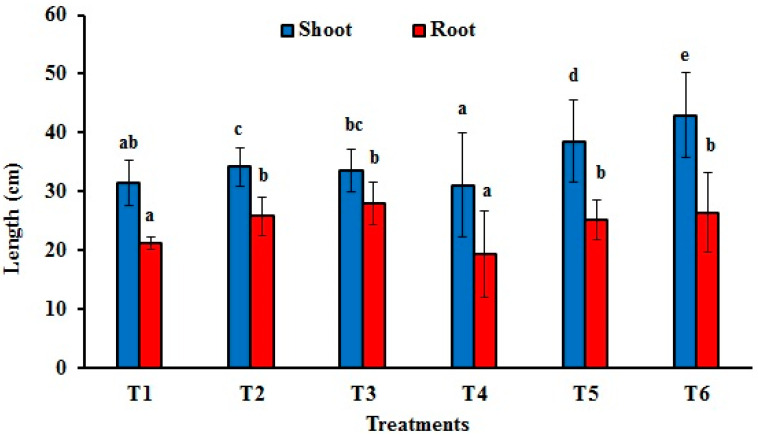
Effect of *Trichoderma afroharzianum* (AEMCTa3 and AEMCTa6) on shoot and root lengths of maize plants infected or uninfected with *Fusarium proliferatum*. Different letters denote significant differences between treatments at a significance level of *p* < 0.05. T1: soil free from fungal inoculum; T2: soil inoculated with *T*. *afroharzianum* AEMCTa3; T3: soil inoculated with *T*. *afroharzianum* AEMCTa6; T4: soil inoculated with *F*. *proliferatum*; T5: soil inoculated with *F*. *proliferatum* + *T*. *afroharzianum* AEMCTa3; T6: soil inoculated with *F*. *proliferatum* + *T*. *afroharzianum* AEMCTa6. The vertical bars indicate the standard deviation of the mean values.

The inoculation of *Fusarium proliferatum* (T4) reduced maize seedlings’ shoot and root fresh weights by approximately 19.1% and 14%, respectively, compared to the control (T1). Their dry weights were also suppressed by 25% and 23.5%, respectively. However, applying *T. afroharzianum* showed an obvious promotion in both fresh and dry weights across all treatments compared to the control (T1) and *F*. *proliferatum* (T4). The maximum shoot fresh weight (17.45 g) and root fresh weight (12.04 g) were observed in T6 and T3 treatments, respectively. The highest shoot dry weight (1.94 g) was estimated in the T6 treatment, while the highest root dry weight (2.18 g) was observed in the T5 treatment (Table 3, Figure 6).

The application of *Trichoderma* species improved the shoot and root lengths of maize plants compared with those of the control (T1) and mitigated the inhibitory effect of *F*. *proliferatum* (Figure 5 and Figure 6).

#### 3.4.3. Photosynthetic Pigments of *Zea mays* Plants

**Figure 7:**
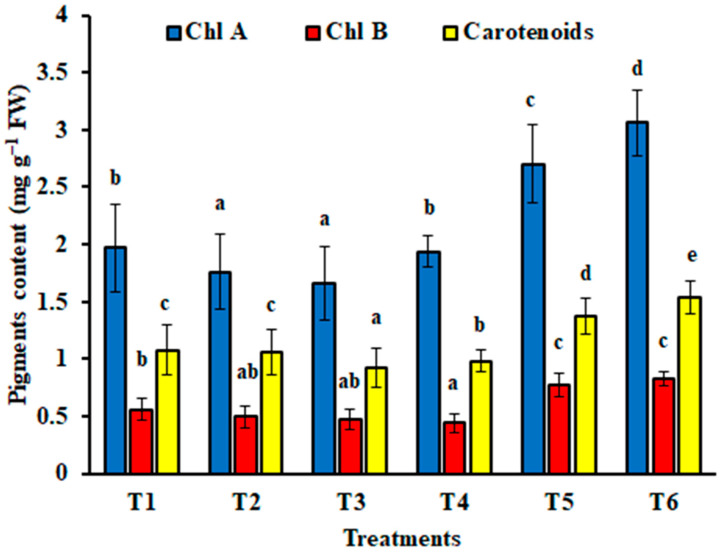
Effect of *Trichoderma afroharzianum* (AEMCTa3 and AEMCTa6) on photosynthetic pigments of maize plants infected or uninfected with *Fusarium proliferatum*. Different letters denote significant differences between treatments at a significance level of *p* < 0.05. T1: soil free from fungal inoculum; T2: soil inoculated with *T*. *afroharzianum* AEMCTa3; T3: soil inoculated with *T*. *afroharzianum* AEMCTa6; T4: soil inoculated with *F*. *proliferatum*; T5: soil inoculated with *F*. *proliferatum* + *T*. *afroharzianum* AEMCTa3; T6: soil inoculated with *F*. *proliferatum* + *T*. *afroharzianum* AEMCTa6. The vertical bars indicate the standard deviation of the mean values.

The effect of *T. afroharzianum* applications on photosynthetic pigments is illustrated in Figure 7. Although *T. afroharzianum* resulted in a decrease in the content of photosynthetic pigments (T2 and T3) when compared to the control (T1), a significant increase was detected in the maize seedlings grown with mixed inoculum of *F*. *proliferatum* and *T*. *afroharzianum* (T5 and T6). *T. afroharzianum* AEMCTa6 had a more beneficial effect on the chlorophyll content of infected maize seedlings than *T. afroharzianum* AEMCTa3 (Figure 7).

### 3.5. Secondary Metabolites Produced by T. afroharzianum AEMCTa3 and AEMCTa6

Thirty-two and twenty-two compounds were detected in *T. afroharzianum* AEMCTa3 and AEMCTa6 extracts, respectively. The identified secondary metabolites were well known for various biological properties, including antimicrobial activities and growth promotion. Notably, 6-Amyl-Alpha-Pyrone and gibberellic acid were among the most abundant compounds, while others such as N-ethyl-1,3-dithioisoindoline and 3-Methyl-2-Phenyl-1H-Indole appeared in lower concentrations. A range of indole-based compounds and other secondary metabolites were also detected, contributing to the potential biocontrol activity of these strains. Full compound names and concentrations are provided in Table 4.

### 3.6. Histological Observations of Zea mays Roots

**Figure 8:**
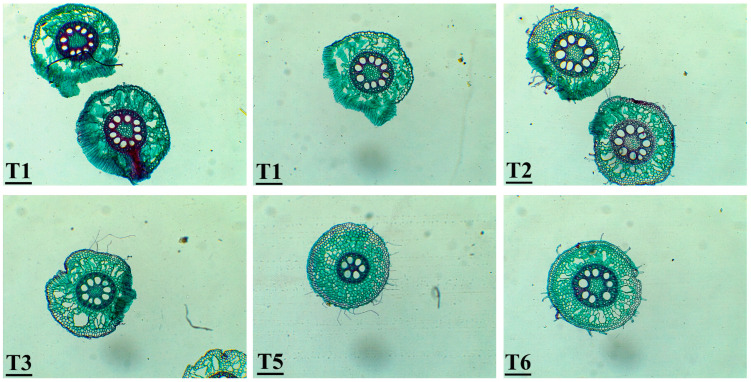
Transverse section of corn roots: T1: soil without fungal inoculum; T2: soil with 0.2% *T. afroharzianum* AEMCTa3; T3: soil with 0.2% *T. afroharzianum* AEMCTa6; T5: soil with 1% *F. proliferatum* + 0.2% *afroharzianum* AEMCTa3; T6: soil with 1% *F. proliferatum* + 0.2% *T. afroharzianum* AEMCTa6. Magnification at 10×.

Based on the histological characteristics of corn roots, many cells were structurally changed compared with the control in response to the root treatments with *Fusarium* and *Trichoderma*. The root hairs appeared in all treatments and were not found in the control. The number of root hairs in T6 was more than that in other treatments. The size of the metaxylem was wider in all treatments compared to the control. T2 and T6 showed metaxylem with a significantly larger size than others (Figure 8).

## 4. Discussion

This study evaluated the morphological characteristics of *Trichoderma* isolates on the PDA and MEA media, with MEA proving to be more effective in highlighting key features [34]. Observed variations in conidial shape, mycelial texture, and growth rate may serve as markers for classification, supporting previous findings [7,35]. Environmental factors like temperature and medium composition significantly influence phenotypic expression, which is important for both taxonomic identification and practical applications in agriculture and industry. The sequences based on the partial *tef-1α* gene were employed to identify *Fusarium* and *Trichoderma* species [36]. The molecular identification of the partial *tef-1α* gene has been widely applied in previous phylogenetic analyses of *Fusarium* and *Trichoderma* species [37,38].

In this study, all *Trichoderma* strains showed an antagonistic effect against *F. proliferatum* with inhibition ranges of 47.65 to 100%. According to Chen et al. [39], seven strains of *Trichoderma* were isolated from the *Radix pseudostellariae* rhizosphere, and their potential to antagonize *F. oxysporum* was assessed in vitro. Among these isolates, the inhibition rates varied, with the highest being 47.91% and the lowest being 16.67%. Elshahawy and Marrez [40] reported that, due to a combination of different responses of antibiosis, mycoparasitism, and competition, the *Trichoderma asperellum* strain showed antagonistic activity against the examined *Fusarium* isolates in the dual-culture experiment. The inhibitory zone and minimum inhibitory concentration values of the crude extracts of *T. asperellum* against the investigated *Fusarium* strains ranged from 7.3 to 19.7 mm and 0.15 to 1.42 mg/mL, respectively.

In this study, we emphasize the potential of AEMCTa3 and AEMCTa6 isolates of *T. afroharzianum* as prospective biocontrol agents against *F. proliferatum*, especially in the context of sustainable agriculture. Their strong antagonistic activity and ability to enhance plant growth parameters suggest a dual role in disease suppression and plant health promotion. These findings further support the growing recognition of *Trichoderma* species as sustainable and eco-friendly alternatives to chemical fungicides in crop protection. The reliability of *Trichoderma* spp. in lowering disease incidence and enhancing the growth of plants is further supported by the similarity of our findings with those of earlier research [41,42]. Moreover, the observed improvements in photosynthetic pigment concentration and biomass agree with studies from Mei et al. [43], Mazungunye and Ngadze [44], Haque et al. [45], and Meddad-Hamza et al. [46], suggesting that these benefits may be broadly applicable to different crops and growth conditions. Chinheya et al. [47] reported that selected *Trichoderma* isolates significantly inhibited *Phytophthora infestans* in vitro and reduced disease symptoms in vivo, including nematode galling and late blight severity. These treatments also enhanced plant physiological criteria such as chlorophyll content and tuber weight, focusing on the roles of *Trichoderma* in disease suppression and growth promotion. Abbod et al. [48] demonstrated that combining *T. harzianum* with plant compost significantly improved maize root biomass under pathogen stress, even surpassing untreated healthy controls. Mitrović et al. [49] reported that *T. harzianum* is an effective biocontrol agent against *F. graminearum* and *Aspergillus flavus* in maize. Field treatments with this bioagent reduced ear rot severity, improved grain yield, and lowered mycotoxin levels. In maize, *Trichoderma asperellum* enhances the plant resistance to pathogens by upregulating the gene expression of plant phytohormones (salicylic acid and jasmonic acid), peroxidase gene, pathogenesis-related protein 5, and allene oxide synthase, effectively reducing *F. graminearum* infection [50]. Helpful fungi like *Trichoderma* can stimulate powerful defense responses in plants. This leads to the production of protective compounds of phenylpropanoids like coumarins and anthocyanins, helping plants survive under stress and resist microbial infection [51].

In this study, various metabolites were identified in the two extracts of *T. afroharzianum*. 6-Amyl-Alpha-Pyrone, produced by *Trichoderma* species, is an eco-friendly, pathogen control and sustainable management tool for future agricultural production [52]. *Trichoderma* species produce antibiotics such as δ-lactone 6-pentyl-α-pyrone (6-PP) [53]. In our study, gibberellic acid was detected, confirming that *Trichoderma* species can produce plant hormones, such as abscisic acid, auxins, cytokinins, ethylene, and gibberellins, all of which are known to enhance plant growth, especially under stressful conditions [54]. The biosynthesis of indole-3-acetic acid produced by the *Trichoderma* stimulates root growth, secondary root production, and root hairs [55]. Phenylacetic acid, 2-(1-adamantyl)ethyl ester, 1-(3-Methylphenyl)-1H-Indole, 1-Methyl-3-phenylindole, 3-Methyl-2-Phenyl-1H-Indole, 2-Phenyl-N-methylindole, and Indolo[2,3-a]quinolizine 17-norcorynan-18-carboxylic acid are derivatives of phenylacetic acid and were identified in the extract of *T. afroharzianum* in this study, and phenylacetic acid was for the first time recorded as one of the secondary metabolites of *Trichoderma*. Nakashima et al. [56] recorded that phenylacetic acid is an aromatic compound characterized by antifungal activity. 1-Hydroxy-4-Nitrobenzene is detected in this study, and the presence of both hydroxy and nitro groups is sometimes associated with antimicrobial activity in aromatic compounds [57]. In this study, 2-Chloro-2-Phenylethylamine was detected at a low concentration. While related chloro- and phenylethylamine derivatives have shown modest antimicrobial activity, their effectiveness largely depends on structural modifications around the phenylethylamine core [58]. 3,5-di-tert-Butylcatechol was also detected at low concentrations in this study. This compound and its derivatives are known for their antimicrobial activity, particularly against bacteria [59]. The compound 4-[4-(4-Bromo-Phenyl)-Thiazol-2-Yl]-Methyl-Amino]-Butyric Acid was detected in notable amounts. Thiazole derivatives with phenyl and bromo substitutions are known for their antimicrobial properties [60]. N-ethyl-1,3-dithioisoindoline showed antimicrobial activity against bacteria like *Bacillus subtilis* and *Klebsiella pneumoniae* strains [61].

The histological analysis of seedling roots revealed that treatment with *Trichoderma* resulted in wider metaxylem vessels and the development of root hairs, compared to untreated control seedlings. The combined physiological and histological analyses confirmed the effectiveness of *T. afroharzianum*, particularly AEMCTa6 (T6), in eliminating the harmful effects of *F. proliferatum* on maize (based on the observed reduction in damping-off incidence from 50% to 0%). T6 not only removed disease symptoms and increased shoot and root biomass but also led to remarkable structural changes in maize roots. T6-treated roots showed increased root hair formation and wider metaxylem vessels as compared to the control, and thus enhanced nutrient uptake and vascular growth and development. These combined results highlight T6 as promoting plant health externally through growth and internally through root tissue development. Harman et al. [62] reported that maize seedlings grown from seeds treated with *Trichoderma harzianum* developed significantly larger roots and shoots compared to those from untreated seeds. The root systems of seedlings treated with *Trichoderma harzianum* were nearly twice as long as those of the control group, with noticeable enhancement in both fine and primary root growth. Root area measurements, which were proportional to root length, indicated that the total root area and volume in treated plants were approximately double those of the controls. While the overall root hair area increased in seedlings treated with *T. harzianum*, the density of root hairs per unit length was higher in the control plants. These distinctions became apparent as early as five days after germination and remained consistent as the plants continued to grow.

*Trichoderma* species have a variety of long-lasting effects on plant development, especially those with substantial rhizosphere competence. They are known to encourage the growth of both roots and shoots, most likely as a result of their direct interactions with plants as well as their function in biological defense. Furthermore, by directly promoting nutrient uptake or by solubilizing soil nutrients, these fungi may promote plant growth [63,64]. No single mechanism can adequately account for their influence in agroecosystems owing to the complexity of their interactions. Nonetheless, there is mounting evidence indicating that *Trichoderma* spp. have a great deal of promise for use in agriculture.

In conclusion, the partial sequencing of *tef-1α* gene was effective in distinguishing *Trichoderma* species, confirming the morphological identification of *T. afroharzianum* and *T. longibrachiatum*. *T. afroharzianum* exhibited strong biocontrol activity against *Fusarium* and significantly promoted maize growth. These findings highlight the potential of *Trichoderma* spp. as sustainable, eco-friendly alternatives to chemical inputs in maize cultivation, contributing to more valuable and environmentally friendly agricultural practices. Future studies on comprehensive metabolomic and transcriptomic analyses guarantee understanding the complex association between *Trichoderma* and plant health to identify specific compounds responsible for the plant growth promotion and show the principal mechanisms, which will help in the development of advanced approaches in sustainable farming.

## Figures and Tables

**Table 1:** Morphological characteristics of *Trichoderma* species on MEA and PDA media after incubation at 28 °C for 7 days.

Morphology and Microscopic Features	*Trichoderma* Species on MEA		*Trichoderma* Species on PDA	
	*T. afroharzianum*	*T. longibrachiatum*	*T. afroharzianum*	*T. longibrachiatum*
Colony appearance	White mycelium colony with greenish ring at center or edges	Gray greenish, sometimes white mycelium with greenish ends	White aerial mycelium with ring and greenish and cottony colony, or white and sparse or grayish green aerial mycelium	Greenish in two rings or one, white mycelium rarely formed, or gray greenish colony in one ring with abundant appearance, rarely produced yellow pigmentation around colony
Conidia width (µm)	2.45–3.63	2.32–3.28	2.35–2.73	2.25–3.76
Conidia length (µm)	2.93–7.58	2.87–9.42	2.83–5.75	2.6–4.89
Conidia shape	Ovate, sub-globose and globose abundant, ellipsoidal rare, smooth	Ellipsoidal-to-ovate abundant, globose rare, smooth.	Ovate, sub-globose and globose abundant, ellipsoidal rare, smooth.	Ellipsoidal-to-ovate abundant, smooth
Shape of phialides	Ampuliform	Lageniform	Ampuliform	Lageniform
Chlamydospores	Present	Absent	Rarely formed	Absent

**Table 2:** The inhibition activity of *Trichoderma* strains against *Fusarium proliferatum*.

Strain Code	Species Name	Source of Isolate	Inhibition% (Mean ± SD, no. = 3)
AEMCTa2	*T. afroharzianum*	Maize seeds, Egypt	47.65 * ± 14.05
AEMCTa3	*T. afroharzianum*	Soil, Italy	100 * ± 0
AEMCTa5	*T. afroharzianum*	Soil, Italy	52.55 * ± 10.75
AEMCTa6	*T. afroharzianum*	Soil, Italy	100 * ± 0
AEMCTl8	*T. longibrachiatum*	Maize seeds, Egypt	53.75 * ± 18.45
AEMCTl9	*T. longibrachiatum*	Maize seeds, Egypt	56.6 * ± 17.3
AEMCTl10	*T. longibrachiatum*	Maize seeds, Egypt	55.5 * ± 11.2
AEMCTl11	*T. longibrachiatum*	Maize roots, Egypt	62.15 * ± 7.25
AEMCTl12	*T. longibrachiatum*	Maize seeds, Egypt	68.9 * ± 1.7
AEMCTl13	*T. longibrachiatum*	Soil, Egypt	63.7 * ± 3
AEMCTl14	*T. longibrachiatum*	Maize seeds, Egypt	52.35 * ± 18.75
AEMCTl1	*T. longibrachiatum*	Maize seeds, Egypt	66.45 * ± 5.75
AEMCTl15	*T. longibrachiatum*	Maize seeds, Egypt	60.6 * ± 15.5
AEMCTl4	*T. longibrachiatum*	Lemon fruit, Egypt	62.8 * ± 12.8
AEMCTl16	*T. longibrachiatum*	Maize seeds, Egypt	58.35 * ± 11.65
AEMCTl17	*T. longibrachiatum*	Lemon fruit, Egypt	62.75 * ± 7.85
AEMCTl18	*T. longibrachiatum*	Maize seeds, Egypt	61.35 * ± 4.75
AEMCTl19	*T. longibrachiatum*	Grape vine, Egypt	56.55 * ± 7.35
AEMCTl20	*T. longibrachiatum*	Grape vine, Egypt	49.5 * ± 21.6
AEMCTl7	*T. longibrachiatum*	Grape vine, Egypt	63.55 * ± 5.35

* Means of inhibition % were significant at *p* < 0.05. ±SD: standard deviations.

**Table 3:** Effect of *Trichoderma afroharzianum* on disease incidence and biomass (fresh and dry weights) of maize plants with or without *Fusarium proliferatum* infection.

Treatments	Treatment Details	Disease Incidence	Fresh Weight (g)		Dry Weight (g)	
			Shoot	Root	Shoot	Root
T1	Soil without fungal inoculum	0 ± 0 ^a^	10.59 ± 2.51 ^a^	6.70 ± 1.06 ^b^	1.245 ± 0.28 ^b^	1.02 ± 0.18 ^b^
T2	Soil with 0.2% *T. afroharzianum* AEMCTa3	5.55 ± 5.6 ^b^	9.63 ± 1.07 ^a^	7.58 ± 0.84 ^c^	1.39 ± 0.16 ^c^	1.15 ± 0.12 ^c^
T3	Soil with 0.2% *T. afroharzianum* AEMCTa6	0 ± 0 ^a^	14.55 ± 1.33 ^b^	12.04 ± 1.09 ^f^	1.68 ± 0.15 ^d^	1.71 ± 0.15 ^e^
T4	Soil with 1% *F. proliferatum*	50 ± 16.66 ^d^	8.57 ± 0.95 ^a^	5.76 ± 0.64 ^a^	0.93 ± 0.10 ^a^	0.78 ± 0.08 ^a^
T5	Soil with 1% *F. proliferatum* + 0.2% *afroharzianum* AEMCTa3	16.66 ± 0 ^c^	16.08 ± 1.79 ^bc^	11.28 ± 1.25 ^e^	1.763 ± 0.19 ^e^	2.18 ± 0.24 ^f^
T6	Soil with 1% *F. proliferatum* + 0.2% *T. afroharzianum* AEMCTa6	0 ± 0 ^a^	17.45 ± 1.94 ^c^	9.43 ± 1.05 ^d^	1.94 ± 0.21 ^f^	1.55 ± 0.17 ^d^

Different letters indicate significant differences among treatments at *p* < 0.05. ±SD: standard deviations.

**Table 4:** Secondary metabolites produced by *Trichoderma afroharzianum* identified by gas chromatography–mass spectrometry (GC-MS) and probability of antimicrobial (A) and/or plant growth promoter activity (P).

Compound No.	Analyte/Parameter	AEMCTa3 Description	AEMCTa6 Description	Activity
1	1-Hydroxy-4-Nitrobenzene	-	Value: 0.943% Retention time: 56.313 min	A
2	2-Butyl-3a,4,7,7a-tetrahydro-1H-isoindole-1,3(2 H)-dione	-	Value: 0.016% Retention time: 82.537 min	-
3	2-Oxo-4-Nitrosomethyl-6-Trifluoro-Methyl-1,2-D ihydropyrimidine	-	Value: 0.779% Retention time: 83.585 min	-
4	4-(3-Chloro-4-Morpholin-4-Yl-Phenyl)-1-(4-Chlo ro-Phenyl)-5-(3,4-Dimethoxy-Phenyl)-4,5-Dihyd ro-1h-[1,2,4]Triazole-3-Carboxylic Acid Ethyl Ester	-	Value: 1.884% Retention time: 26.208 min	-
5	6-Ethyl-2,3-dihydro-2,7-dimethyl-5-oxo-5H-Oxa zolo [3,2-a]pyridine-8-carbonitrile	-	Value: 0.248% Retention time: 44.702 min	-
6	9,10-Dehydro-6-Desoxy-Indolinocodeine	-	Value: 1.004% Retention time: 56.785 min	-
7	Beta.-Dihydroagarofuran	-	Value: 0.411% Retention time: 52.167 min	-
8	Decane	-	Value: 0.466% Retention time: 16.667 min	-
9	Ethanediimidic Acid Dihydrazide	-	Value: 1.228% Retention time: 41.105 min	-
10	Heptadecane	-	Value: 0.724% Retention time: 60.110 min	-
11	Pentadecane	-	Value: 0.353% Retention time: 58.163 min	-
12	Tetracosane	-	Value: 0.466% Retention time: 46.067 min	-
13	Triacontane	-	Value: 0.693% Retention time: 55.983 min	-
14	5-(1,3,5-trimethyl-4-pyrazolyl)amino-1,2,4-Triaz ol-3-amine	Value: 1.125% Retention time: 63.922 min	-	-
15	(.+.)-1,4,5,6,7,8-hexahydro-6,6,7,7,8,8-hexam ethyl-2,4-diphenyl-2H-cyclopent[D][1,2]oxazepi ne	Value: 0.382% Retention time: 62.913 min	-	-
16	(+)-5-(1-Acetoxy-1-methylethyl)-2-methyl-2-cy clohexen-1-one semicarbazone	Value: 0.417% Retention time: 53.967 min	-	-
17	(5,10)-(7,8)-Dioxirane derivative of .alpha.-4-phenyl-1,2,4-triazolin-3,5-dione adduct of vitamin d3 (Isomer A)	Value: 0.592% Retention time: 65.128 min	-	-
18	(5,10)-(7,8)-Dioxirane derivative of .alpha.-4-phenyl-1,2,4-triazolin-3,5-dione adduct of vitamin d3 (Isomer B)	Value: 0.256% Retention time: 46.302 min	-	-
19	(5-Ethoxycarbonylamino-2,6-dimethylpyridin-3- yl)carbamic acid, ethyl ester	Value: 0.256% Retention time: 46.602 min	-	-
20	1-(2-Chlorophenyl)-1,2,3,4-tetrahydro-3-metho xycarbonyl-9H-indolo[2,3-c]pyridine	Value: 1.644% Retention time: 65.806 min	-	-
21	1-(3-Methylphenyl)-1H-Indole	Value: 1.328% Retention time: 63.663 min	-	P
22	1-(4-Methyl-2-[(trimethylsilyl)oxy]phenyl)ethano ne	Value: 1.159% Retention time: 60.038 min	Value: 0.410% Retention time: 96.684 min	-
23	1-(5-Methyl-2-[(Trimethylsilyl)Oxy]Phenyl)Etha none	Value: 1.658% Retention time: 71.188 min	-	-
24	1-Methyl-3-phenylindole	Value: 0.273% Retention time: 52.178 min	-	P
25	2-(5-Adamantan-1-yl-[1,2,4]oxadiazol-3-yl)-pyri dine	Value: 0.232% Retention time: 47.923 min	Value: 0.058% Retention time: 71.443 min	-
26	2,4-Dimethylbenzo[h]quinoline	Value: 1.004% Retention time: 77.869 min	-	-
27	2-Amino-4,4,6,6-tetramethyl-4,6-dihydro-thieno [2,3-c]furan-3-carbonitrile	Value: 1.658% Retention time: 48.985 min	-	-
28	2-Chloro-2-Phenylethylamine	Value: 0.749% Retention time: 67.672 min	-	A
29	2-Phenyl-N-methylindole	Value: 1.506% Retention time: 59.547 min	-	P
30	3,5-Dimethyl-2,6-bis(trimethylsiloxy)pyridine	Value: 1.189% Retention time: 68.454 min	-	-
31	3,5-Ditert-Butyl-4-Hydroxy-2,4-Cyclohexadien- 1-One	Value: 1.129% Retention time: 72.967 min	-	-
32	3,5-di-tert-Butylcatechol	Value: 0.354% Retention time: 66.431 min	Value: 0.115% Retention time: 97.732 min	A
33	3-Amino-2-Hydroxy-5-Sulfobenzoic Acid	Value: 1.157% Retention time: 75.817 min	-	-
34	3-Methyl-2-Phenyl-1H-Indole	Value: 0.259% Retention time: 45.514 min	-	P
35	4-[[4-(4-Bromo-Phenyl)-Thiazol-2-Yl]-Methyl-A mino]-Butyric Acid	Value: 6.944% Retention time: 69.462 min	Value: 0.051% Retention time: 82.472	A
36	4-Chloro-2-trifluoromethylbenzo[h]quinoline	Value: 0.885% Retention time: 79.213 min	-	-
37	5-(p-Aminophenyl)-4-(p-tolyl)-2-thiazolamine	Value: 0.654% Retention time: 62.095 min	-	-
38	5,5′,6,6′,8,8′-Hexamethoxy-2,2′-Dimethyl-[9,9′-Bi-4h-Naphtho[2,3-B]Pyran]-4,4′-Dione	Value: 1.380% Retention time: 75.115 min	-	-
39	6-Amyl .Alpha. Pyrone	Value: 1.552% Retention time: 64.814 min	Value: 35.552% Retention time: 42.412 min	P
40	8,11-Epoxy-9,12-Ethano-11,15-Methano-11h-[1,8]Dioxacycloheptadecino[4,3-b]Pyridine, Evonine deriv.	Value: 0.371% Retention time: 56.772 min	-	-
41	8-Methylisothiazolo[4,5-C]-2,1,3-Benzothiadiaz ole	Value: 1.544% Retention time: 69.840 min	Value: 0.065% Retention time: 81.903 min	-
42	Gibberellique Acid	Value: 26.420% Retention time: 42.399 min	Value: 10.760% Retention time: 96.496 min	P
43	Indolo[2,3-a]quinolizine, 17-norcorynan-18-carboxylic acid deriv.	Value: 0.287% Retention time: 78.887 min	-	P
44	N-ethyl-1,3-dithioisoindoline	Value: 0.259% Retention time: 47.244 min	Value: 0.304% Retention time: 83.145 min	A
45	Phenylacetic acid, 2-(1-adamantyl)ethyl ester	Value: 0.526% Retention time: 78.222 min	Value: 0.380% Retention time: 84.102 min	P

(-) means the compound was not detected in the isolate.

## Data Availability

Accession numbers are inserted in the manuscript (in the phylogenetic tree), and they are available in GenBank: https://www.ncbi.nlm.nih.gov/nucleotide/ (accessed on 24 July 2025).
